# Supplementary material for: Overexpression of UBA5 in Cells Mimics the Phenotype of Cells Lacking UBA5
Source: Int J Mol Sci. 2022 Jul 4;23(13):7445. doi: 10.3390/ijms23137445 (PMC9267032; doi:10.3390/ijms23137445)
Supplement: Supplementary file 1 [file ijms-23-07445-s001.zip › ijms-1770179-supplementary.pdf]

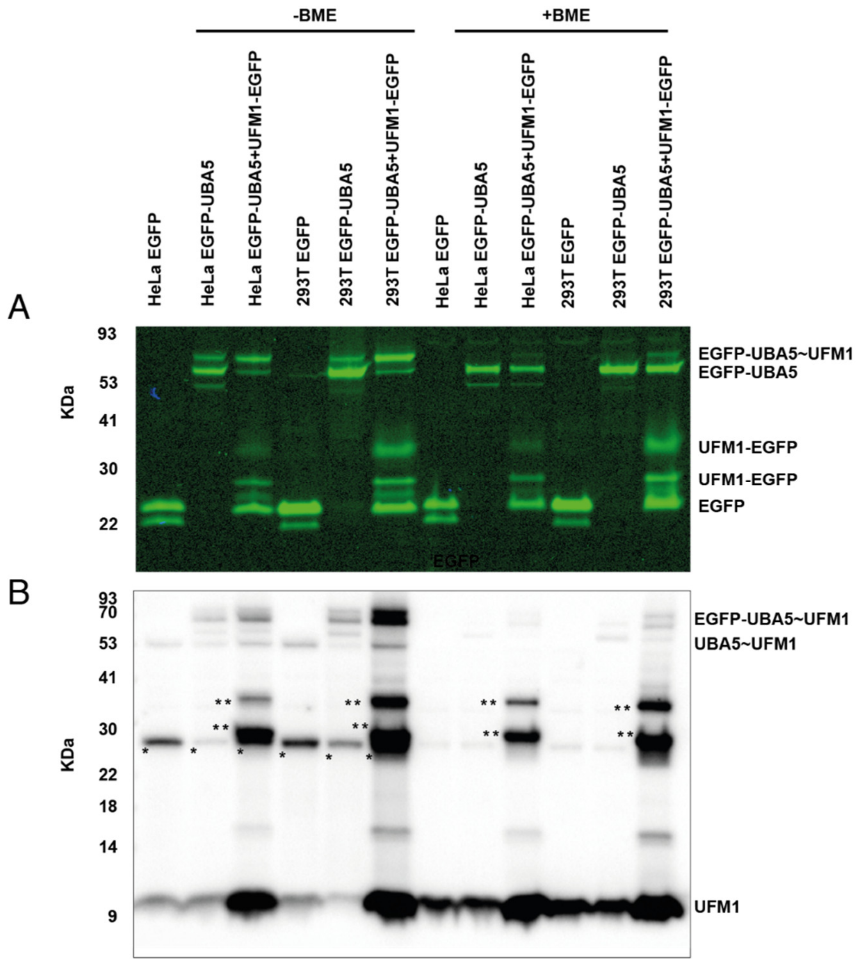

Supplementary Figure S1. (**A**) Fluorescent gel showing the expression level of EGFP-UBA5 alone or co-expressed with UFM1-EGFP in the indicated cell lines. EGFP has two bands in the SDS-PAGE (lane 1). Overexpression of UFM1-EGFP yields two bands corresponding to UFM1-EGFP and two bands corresponding to free EGFP. Free EGFP is obtained by the endogenous UFM1 protease (UFSP2) that cleaves UFM1-EGFP. (**B**) Western blot analysis of the above fluorescence gel. The level of free UFM1 increases in the lanes possessing overexpression of UFM1-EGFP. * indicates charged UFC1 (UFC1~UFM1) that disappears in the presence of BME; ** indicates UFM1-EGFP bands.

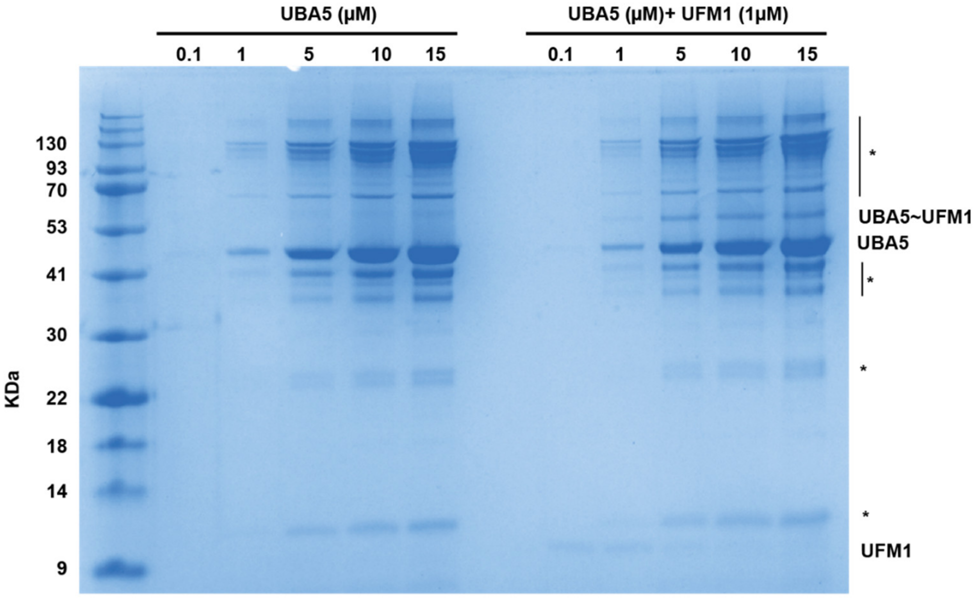

Supplementary Figure S2. Coomassie-stained SDS-PAGE showing the purity of UBA5 at different concentrations (left) or together with UFM1 (right). The band corresponding to UBA5~UFM1 exists only in the presence of UFM1. Contaminations that arrive with UBA5 are indicated with an asterisk. This experiment was executed without the addition of ATP. The residual ATP that is associated with the recombinant UBA5 allows the charging of UBA5.

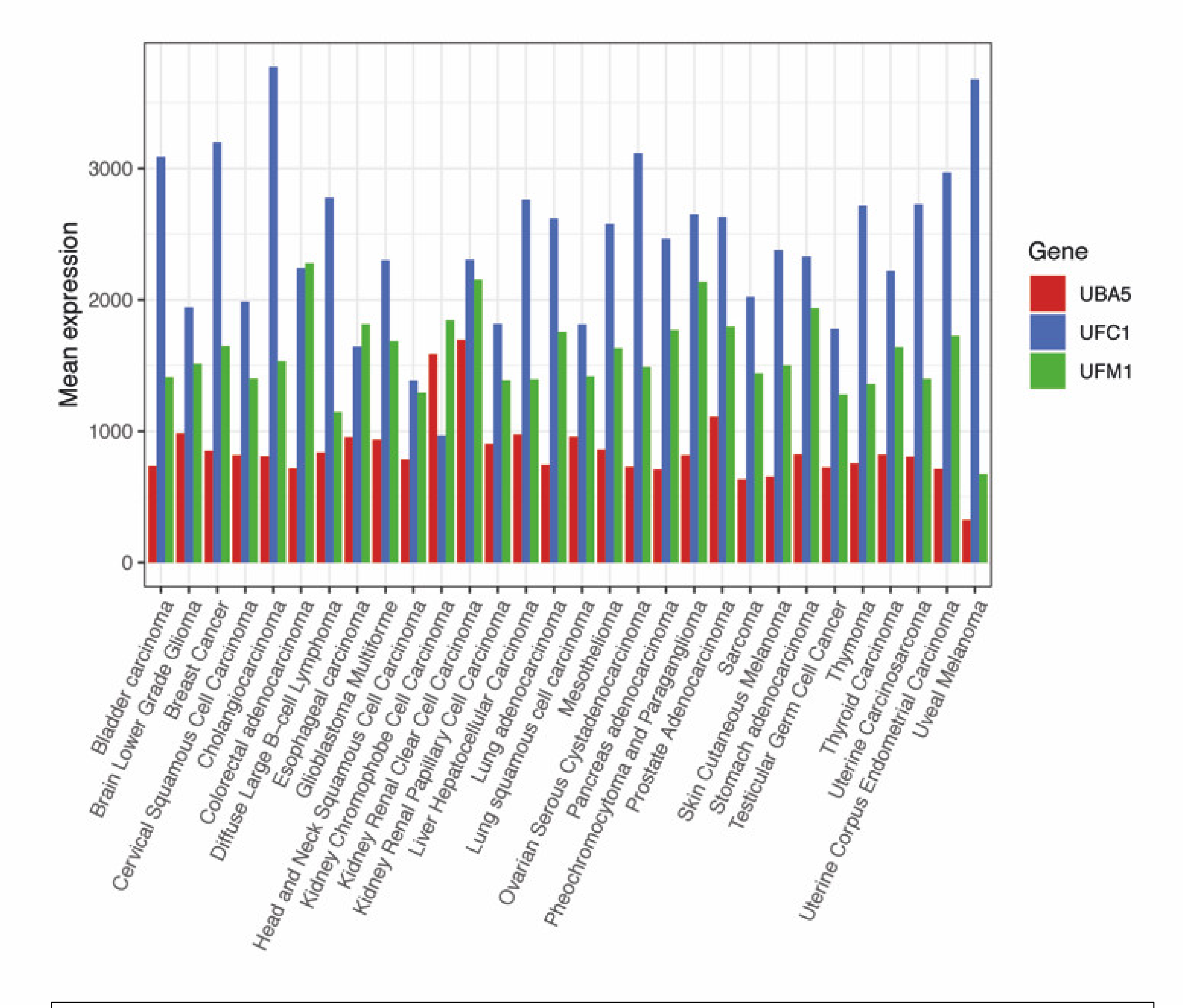

Supplementary Figure S3. RNA expression level of the indicated genes in different cancerous cells. Data obtained from cBioProtal (https://www.cbioportal.org).
